# Coronary Angiography Using Noninvasive Imaging Techniques of Cardiac CT and MRI

**DOI:** 10.2174/157340308786349444

**Published:** 2008-11

**Authors:** Shun Kohsaka, Amgad N Makaryus

**Affiliations:** 1 Department of Cardiology, Keio University, School of Medicine, Tokyo, Japan, North Shore University Hospital, Manhasset, NY, USA; 2 Department of Medicine, Division of Cardiology, North Shore University Hospital, Manhasset, NY, USA

**Keywords:** Coronary angiography, cardiac computed tomography, magnetic resonance imaging.

## Abstract

Noninvasive coronary angiography has become an important imaging tool in the evaluation of patients with and at risk for coronary artery disease (CAD). Multidetector computed tomographic (MDCT) angiography offers excellent negative predictive value (≥95%) for the absence of coronary artery disease and has shown promising results in evaluating allograft vasculopathy, bypass grafts, and degenerative aortic valve disease. A single MDCT scan in the emergency department is valuable in ruling out both cardiac and noncardiac causes of acute chest pain. Cardiac magnetic resonance (MR) currently lacks the spatial resolution of MDCT limiting its assessment of the coronary vasculature, but the proximal coronary arteries can be evaluated along with myocardial function and viability without exposure to contrast dye or ionizing radiation. In addition, MR imaging also has great potential for characterizing coronary plaques, as well as following their progression and regression.

## INTRODUCTION

Invasive coronary angiography is the gold standard for establishing the presence, location, and severity of coronary artery disease (CAD) [1,2]. While invasive coronary angiography provides excellent spatial and temporal resolution for the visualization of the coronary arterial tree for catheterbased or surgical interventions, this technique is invasive, costly [2] and associated with a small but definite risk of morbidity (1.5%) and mortality (0.15%) [3,4]. Therefore, a convenient, non-invasive alternative method for coronary angiography can provide significant clinical and economic benefits for most patients with and at risk for coronary artery stenosis [5]. 

Noninvasive cardiac imaging is evolving rapidly. Multi-detector cardiac computed tomography (MDCT) and magnetic resonance (MR) imaging have become the preeminent modalities for the assessment of coronary artery atherosclerosis. Once used primarily as research tools, these modalities are increasingly being used in routine clinical practice, particularly for direct imaging of the coronary vasculature. A review of the latest evidence is presented with respect to these evolving imaging modalities in the field of cardiology.

## CORONARY CT ANGIOGRAPHY (FIGS. 1 AND 2)

Contemporary multislice/multidetector CT (MSCT/MDCT) techniques, which allow noninvasive evaluation of coronary arteries and bypass grafts, continue to evolve as alternatives to invasive coronary angiography. Modern MDCT systems can provide electrocardiogram-gated acquisition with adequate temporal resolution (100-220ms) and the submillimeter spatial resolution needed to visualize the lumen of the coronary arteries. More recent modern dual source systems have even improved temporal resolution of 85 ms. Currently, 64-slice MDCT has a spatial resolution in the range of 0.6-1.0 mm in all three dimensions (compared with 0.3 mm for conventional angiography). A spatial resolution of 1.0 mm is sufficient for imaging most of the coronary vascular tree, except for distal and calcified segments that require a spatial resolution of 0.5 mm [6].

Numerous studies have evaluated the potential of CT coronary angiography to define focal coronary stenoses (Table **1**). MDCT has shown reliability for ruling out disease in nondiseased patients and is superior to MR for detecting significant atherosclerotic lesions [6]. In selected patients referred for invasive coronary angiography, the sensitivity of 16- and 64-slice CT coronary angiography was 90% or higher in most studies after exclusion of arteries whose image quality was considered to be subdiagnostic. 64-slice MDCT also has shown value in correctly identifying the absence of any atheromatous plaque among patients deemed to be at intermediate-to-high risk of CAD, with a negative predictive value >95% in most recent studies. In addition, as the number of detectors has increased, so has the number of coronary artery segments that can be evaluated. In a recent meta-analysis, 78%, 91%, and 100% of segments could be evaluated with an 8-, 16- and 64-slice MDCT, respectively [7]. 

Leschka *et al.* [8] presented the first study exploring the diagnostic performance of 64-slice CT coronary angiography. They evaluated all coronary segments 1.5 mm and reported a high sensitivity and specificity for detecting significant lesions. Their overall sensitivity for classifying stenoses was 94%, specificity was 97%, positive predictive value was 87%, and negative predictive value was 99% [8]. Raff *et al.* [9] studied 70 consecutive patients, and found that of the 1,065 coronary segments identified, 935 (88%) could be evaluated, and the specificity, sensitivity, and positive and negative predictive values for the presence of significant stenoses identified by 64-CT in comparison to CA were: by segment (n = 935), 86%, 95%, 66%, and 98%, respectively; by artery (n = 279), 91%, 92%, 80%, and 97%, respectively; by patient (n = 70), 95%, 90%, 93%, and 93%, respectively [9]. 

The CATSCAN trial [10], a multicenter 16-detector CT trial included eleven participating sites that prospectively enrolled 238 patients who were clinically referred for nonemergency coronary angiography from June 2004 through March 2005. In their patient-based analysis, the sensitivity for detecting patients with at least 1 positive segment with >50% stenosis was 98%; specificity, 54%; positive predictive value, 50%; and negative predictive value, 99%. More recently, results of the Coronary Evaluation Using Multi-detector Spiral Computed Tomography Angiography using 64 Detectors (CorE-64 trial) [11], the first multicenter trial assessing 64-detector CT scanning, reported a sensitivity of 85% and a specificity of 90% compared with the gold-standard of invasive angiography in a groups of 291 patients.

Although these applications of cardiac MDCT are promising, there is still room for improvement in coronary MDCT image acquisition and post-processing techniques. Current technological limitations still prevent exact quantification of the degree of stenosis and reliable visualization of all small segments. In addition, image quality is compromised when the heart rate is too rapid, the patient is morbidly obese (>350 lbs), or the cardiac rhythm is irregular because of atrial fibrillation, frequent premature atrial or ventricular contractions, or exaggerated sinus arrhythmia. Importantly, MDCT detects significant CAD with excellent accuracy in patients with complete left-bundle branch block (LBBB) [12]. LBBB most commonly correlates strongly to age, associated with atherosclerotic coronary artery disease, and increases risk of cardiac mortality. Previously, non-invasive stress tests have limited performance in subjects with LBBB but MDCT is a robust tool to act as a filter in this setting to avoid unnecessary invasive diagnostic procedures [12].

Also, calcium is a frequent feature of the coronary arteries (found in 70-80% of the population), and complete assessment by MDCT can be hindered by dense, focal calcium deposits in the vessel wall, leading to an overestimation of the severity of stenosis. With so-called “calcium blooming”, dense calcification often preclude assessment with MDCT and such lesions are common in patients with advanced CAD. Therefore, patient selection is crucial.

Finally, intravenous contrast and radiation limit the use of MDCT. The typical intravenous contrast dose is 70-100 cc, and the usual radiation dose is 10-15 mSv [13]. The radiation dose is equivalent to what is absorbed during a stress nuclear perfusion examination and nearly twice as high as conventional coronary angiography without ventriculography or graft imaging. The risk of malignancy and other radiation-related complications is uncertain with these doses, however researchers using dosimetry models have raised concerns regarding exposure [14]. Newer prospectively gated “step and shoot” methods have significantly decreased radiation dose by only turning the radiation tube on during a prespecified period of the cardiac cycle for vessel imaging at end diastole. This technique can bring radiation dose to levels less that 5 mSv [15].

Of note, new directions are being explored as the MDCT technology matures. The 256- and 320-detector MDCT are currently under investigation. These newer scanners will obtain volumetric data with wide “whole-heart/single cardiac cycle” coverage in a single rotation and allow adequate visualization of the cardiac chambers and coronary arteries by cine scan imaging. These characteristics are very promising with respect to solving many of the current limitations of MDCT including motion artifcats and misregistration banding artifacts due to the inclusion of multiple cardiac cycles, and may provide a solution to the problems of current MDCT modality [16-20]. Kido *et al.* [16] showed the ability of the 256-detector row four-dimensional CT to assess coronary arteries and cardiac function using data from a 1.5 s acquisition without the presence of any banding artifacts. Rybicki *et al.* [19] have reported their initial experience with 320-detector cardiac CT. Their initial 320-detector row coronary CT images showed consistently excellent quality and iodinated contrast opacification. 

### Specific Indications

Widespread use of MDCT in broad clinical populations without specific indications can lead to further unnecessary testing and escalating costs [21]. No official guidelines on the use of either modality for cardiac applications have been released but appropriateness criteria [22] have been published as an intermediate step to avoid unnecessary and inappropriate testing. The appropriateness criteria focuses on 39 CT and 33 MR indications identified by the panel as encompassing the majority of cases referred for each of the modalities. Examples of highest-scoring indications included evaluation of intra- and extracardiac structures using cardiac CT or, in the case of suspected coronary anomalies, CT angiography. Lowest-scoring indications, given an "inappropriate" ranking, included evaluation of patients with a high pretest probability of CAD based on risk factors or results of other tests. 

Limited information exists regarding the prevalence of clinically significant incidental unsuspected findings in patients undergoing MDCT. This technique frequently detects clinically occult and potentially life-threatening cardiac (eg. ventricular aneurysm, intramural thrombus) or noncardiac (eg. lung cancer) abnormalities and all physicians interpreting MDCT should be cognizant of these finding. Lastly, long-term studies to determine the predictive prognostic value of MDCT are also necessary, similar to what has been done with electron-beam CT and coronary artery calcification [23]. Still, several specific situations have emerged beyond simple coronary artery imaging in which MDCT may be particularly valuable.

### Bypass Graft Evaluation

Most early (<1 month) graft occlusion, which occurs in up to 10% of patients, is attributable to mechanical causes, whereas the late (5-10 years) stenosis or occlusion that occurs in the majority of grafts results from an accelerated atherosclerotic process. Because many vein graft occlusions are asymptomatic, being able to identify early saphenous vein graft degeneration using CT coronary angiography may allow earlier intervention when graft patency makes revascularization feasible. Bypass grafts are excellent targets for visualization with MDCT because of their reduced overall motion and their large lumens. Graft diameter typically ranges from 4 to 6 mm throughout the conduit, whereas the native vessel can taper to a diameter as small as 1 mm in the distal portion. However, limitations in the visualization of distal anastomosis sites and segments with adjacent clips exist. In a study by Schlosser *et al.*, [24] MDCT showed good accuracy in assessing graft patency, with a sensitivity of 96% and a specificity of 95%. However, it was less well suited to evaluating areas adjacent to surgical clips and the distal bypass anastomosis could not be visualized in nearly 25% of cases. Segments with adjacent clips can also be problematic.

### Cardiac Allograft Vasculopathy

Because noninvasive functional tests have traditionally lacked adequate sensitivity and specificity, conventional coronary angiography is the current gold standard for the serial detection and follow-up of cardiac allograft vasculopathy in heart transplant patients. MDCT may offer a non-invasive alternative. In a series of 53 patients who underwent both routine invasive coronary angiography and MDCT, MDCT’s sensitivity and specificity for detecting coronary stenoses >50% were 83% and 95%, respectively [25]. It was felt that MSCT may offer an advantage over conventional coronary angiography by showing coronary wall thickening as well as luminal narrowing. visual assessment in this situation was noted to be limited by small caliber vessels, tachycardia, and the presence of stents.

### Computed Tomography Scans for the Assessment of Chest Pain in the Emergency Department

The possibility of using MDCT for comprehensive assessment of cardiac and noncardiac causes of chest pain in patients presenting to the emergency room is being evaluated. In particular, a single MDCT scan could be used to rule out coronary artery disease, pulmonary embolism, and aortic dissection. A feasibility study of MDCT in evaluating cardiac and noncardiac causes of acute chest pain in 69 patients presenting to the emergency department [26] showed that MDCT evaluation was comprehensive and produced a false-negative rate of only 3%. Further investigation is needed to determine whether and how patients with low-to-intermediate risk can be triaged effectively using a MDCT algorithm.

Goldstein *et al.* [27] randomized patients presenting to the emergency department with acute chest pain to MDCT (n = 99) versus standard of care (n = 98). The MDCT patients with minimal disease were discharged; those with stenosis >70% underwent catheterization, whereas cases with intermediate lesions or non-diagnostic scans underwent stress testing. Both approaches were found to be 100% safe. The MDCT alone immediately excluded or identified coronary disease as the source of chest pain in 75% of patients, including 67 with normal coronary arteries and 8 with severe disease referred for invasive evaluation. The remaining 25% of patients required stress testing, owing to intermediate severity lesions or non-diagnostic scans. During the index visit, MDCT evaluation reduced diagnostic time compared with standard of care (3.4 h *vs*. 15.0 h, p < 0.001) and lowered costs ($1,586 *vs*. $1,872 p < 0.001). Further, MDCT patients required fewer repeat evaluations for recurrent chest pain (MDCT, 2 of 99 (2.0%) patients *vs*. no MDCT, 7 of 99 (7%) patients; p = 0.10) [27].

This strategy of so-called “triple rule out” protocol (to exclude obstructive coronary artery disease, pulmonary embolism, and aortic dissection simultaneously) in the emergency room is currently investigated. Generally, these protocols are not supported by the guidelines and remains “uncertain” in the appropriateness criteria [22]. Further validation studies are needed to clarify whether patients can be appropriately discharged after negative initial enzyme test and/or no ST-segment changes [28].

### Plaque Characterization (Fig. 2)

MDCT can provide valuable quantitative information on coronary atherosclerotic plaques in an area of research. Specifically, based on plaque Hounsfield unit (HU) intensity, plaques may be categorized as calcified plaques (“hard”), fatty plaques (“soft”), fibrous plaques, or fibrofatty plaques. In terms of quantitative plaque volume measurement, its results correlate highly with those of intravascular ultrasonography [29,30]. In a recent study [29], 41 proximal coronary segments imaged using IVUS, sensitivity and specificity were 95% and 91%, respectively, for calcified plaque, and 91% and 89%, respectively, for noncalcified plaque. Leber *et al.* [30] studied 59 patients scheduled for invasive angiography due to stable angina pectoris. A further subset of 18 patients had intravascular ultrasound (IVUS) of 32 vessels performed as part of the catheterization procedure. The overall correlation between the degree of stenosis detected by quantitative coronary angiography compared with 64-CT was r = 0.54. Sensitivity for the detection of stenosis <50%, stenosis >50%, and stenosis >75% was 79%, 73%, and 80%, respectively, and specificity was 97%. In comparison with IVUS, 46 of 55 (84%) lesions were identified correctly. Plaque and lumen areas derived by CT correlated well with IVUS, however, the results were limited by the insufficient ability of CT to exactly quantify the degree of stenosis despite excluding patients with atrial fibrillation, coronary artery calcification, stenting, and bypass surgery [30]. 

**Fig. (2):**
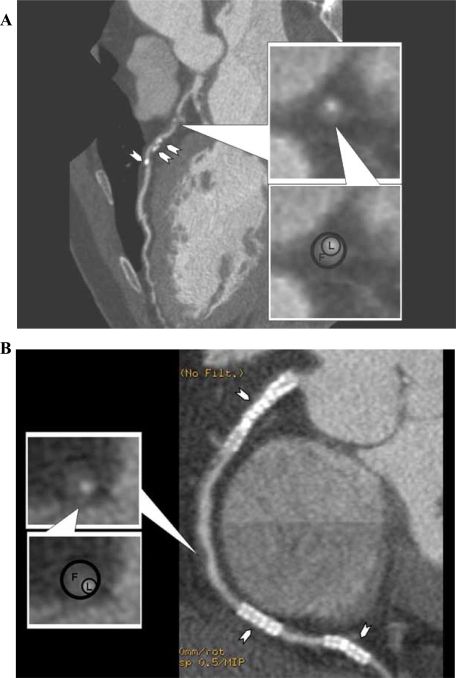
(**A**) 64-slice MDCT curved multiplanar reformatted images from a 72 year old man with a history of coronary artery disease and hypercholesterolemia showing the left anterior descending artery with calcification and fatty plaque in its proximal to mid portion (shown in cross section in the inset images; L=lumen [~100 HU]; F=fatty plaque [40-70 HU]). Arrowheads denote areas of punctuate calcification (120-140 HU). (**B**) Curved multiplanar images from the same patient showing three patent coronary stents (arrowheads) as well as fatty plaques in the mid portion of the right coronary artery (shown in cross section in the inset image; L=lumen [~100 HU]; F=fatty plaque [40-50 HU]).

On the other hand, MDCT cannot yet provide specific qualitative plaque information obtained by MR imaging. CT differentiates plaque composition by Hounsfield unit value and there is a large overlap between plaque types (figure **2**).In particular, differentiation between groups of fatty, fibrous, or fibrofatty plaques can be limited [31-33]. 

## CORONARY MR ANGIOGRAPHY (FIG. 3)

Cardiac MR allows assessment of proximal coronary anatomy, global and regional cardiac function, cardiac volumes, and myocardial viability [34] without exposing patients to intravenous contrast or ionizing radiation. Individually, various cardiac MR techniques have shown promise as alternatives to established noninvasive tools for detecting coronary stenosis and myocardial infarction. Coronary MR angiography is still technically challenging for the assessment of the presence and severity of coronary stenosis owing to small arterial size, tortuosity, complex anatomy, and cardiac and respiratory motion. 

Although there are limitations in the assessment of luminal stenosis, MR is highly efficacious for the evaluation of the course of anomalous coronary arteries. The relationship between the great vessels and the course of coronary arteries is better depicted by MR than by conventional coronary angiography because of the three-dimen-sional ability of MR in comparison with two-dimensional x-ray projections with overlapping structures.

Currently, coronary magnetic resonance angiography (MRA) sequences have resolutions on the order of 1.0 mm. The overall sensitivity and specificity of MRA is as high as 90% for proximal and mid-coronary artery disease (Table **2**). However, coronary MRA is used much less frequently than MDCT angiography to detect CAD because coronary MRA has a longer scan time and lower spatial resolution (1.2-1.4 mm) than MDCT. Current MDCT technique offers the highest spatial resolution available for noninvasive coronary angiography, and the diagnostic performance of MDCT seems to be superior to that of MRI. The most promising MRA technique currently seems to be whole-heart acquisition, in which the entire heart is scanned in a fashion similar to that used in cardiac CT protocols [35]. Other methods that have been investigated include the use of intravascular gadolinium-based agents and 3-dimensional acquisition strategies [36].

Kim *et al.* [37] performed the first multicenter trial where 109 subjects were evaluated for CAD by the free-breathing 3D technique. Lesions in the left main coronary artery in patients with three vessel disease could be identified with some certainty in a limited number of patients. A sensitivity of 83% was reported for this technique. However, this technique was limited to evaluation of proximal and middle segments only. In this study a total of 636 of 759 proximal and middle segments of coronary arteries were interpretable. The study also stated that major limitations with the 3D technique are the relatively longer time (average 70 minutes), low specificity (42%), and low overall diagnostic accuracy (72%). 

Nikolaou *et al.* [38] evaluated 20 patients who had undergone contrast enhanced computed tomography (EBCT) and navigator echo-based coronary MRA with retrospective gating. The results were compared with conventional coronary angiography. The sensitivity and specificity for detecting significant stenoses with coronary MRA were 79% and 70%, respectively, and with EBCT the sensitivity and specificity were 85% and 77%, respectively. The low sensitivity with 3D coronary MRA was attributed to inadequate synchronization during the end-expiration phase [38]. 

### Plaque Characterization

Magnetic resonance imaging has technical limitations that make it more challenging for plaque volume measurements, but it has great potential for noninvasive quality assessment, using a variety of sequencing techniques (eg, T1, T2, fat saturation) [39,40]. In addition to being used to research study atherosclerotic plaques in the human carotids and aorta [41], MR appears particularly promising for identifying vulnerable coronary plaques [42]. Coronary arteries are relatively deeply located and create motion artifact, but in a study using a porcine model of CAD, MR imaging was found to sufficiently differentiate among fibrocellular, lipid-rich, and calcified coronary plaques, and its findings correlated with histopathologic findings [43]. For more precise quantification, contrast agents that target specific molecules (eg, adhesion molecules) or other substances are being developed [44,45]. In animal models, MR has also been a powerful tool in serially investigating *in vivo* the progression and regression of atherosclerotic lesions [46]. Given the rapid development of this field, the ability to identify, aggressively treat, and serially monitor patients with high-risk plaques will probably improve significantly in the near future.

## CONCLUSION

Despite existing limitations, there is an important segment of the population in whom noninvasive imaging could provide coronary anatomic information with sufficient diagnostic quality. Furthermore, various noninvasive techniques offer potential advantages over traditional invasive coronary angiography, such as characterizing coronary plaque, providing both structural and functional information about the left ventricle and heart valves, and not exposing patients to the risk of vascular injury. On the horizon, combined CT and MR imaging may provide information not available from other imaging modalities, including lesion localization along with structural and biological plaque characterization. 

## Figures and Tables

**Fig. (1):**
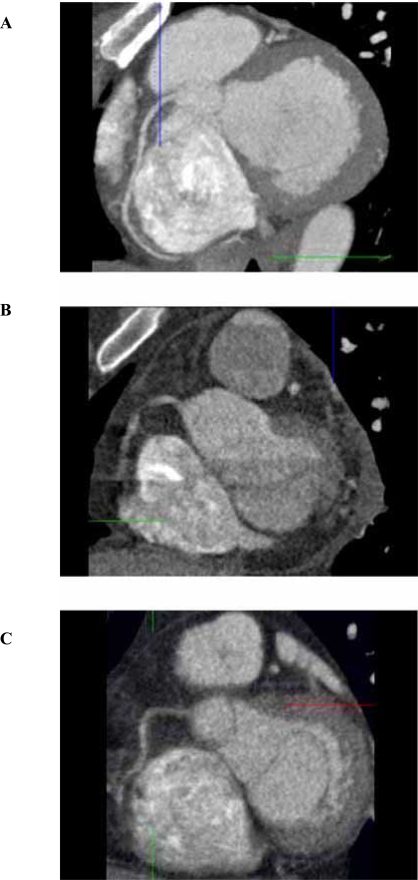
64-slice MDCT images of normal patient with heart rate of 52 bpm (**A**), heart rate of 70 bpm (**B**), and obese patient with body mass index of 36 (**C**). Note in (**A**), the right coronary artery (RCA) is adequately evaluated in this oblique tomographic section. In (**B**), misregistration artifacts due to suboptimal heart rate preclude adequate visualization of the RCA, and in (**C**), adequate assessment of the RCA is precluded due to suboptimal imaging in an obese patient.

**Fig. (3):**
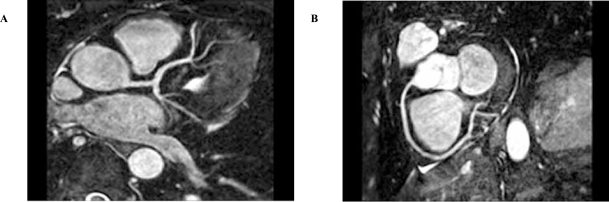
3D breathhold and contrast-enhanced MR angiogram images of the left coronary artery (**A**) and the right coronary artery (**B**).

**Table 1.** Computed Tomographic Angiography of the Coronary Arteries

Technique	Reference	# Patients	Sensitivity	Specificity	Negative Predictive Value
16-slice MDCT (segments >2mm)	Nieman *et al.* Circ 2002; 107: 664	59	86%	95%	97%
	Ropers *et al.* Circ 2002; 107: 664	77	92%	93%	97%
	Mollet *et al.* JACC 2004; 43: 2265	128	95%	92%	98%
	Mollet *et al.* JACC 2005; 45: 128	51	98%	95%	99%
	Hoffmann *et al.* Circ 2004; 110: 2638	103	98%	95%	99%
16-slice MDCT (all-segment analysis)	Kuettner *et al.* JACC 2004; 44: 1230	60	97%	72%	97%
	Kuettner *et al.* JACC 2005; 45: 123	72	92%	82%	97%
	Schuijf *et al.* AJC 2005; 95: 571	45	91%	93%	98%
	Garcia *et al.* JAMA 2006; 296: 4	238	89%	65%	99%
64-slice MDCT	Raff *et al.* JACC 2005; 46: 552	84	86%	95%	98%
	Mollet *et al.* Circ 2005; 112: 2318	52	95%	99%	99%
	Leber *et al.* JACC 2005; 47: 672	59	97%	80%	99%
	Leschka *et al.* EHJ 2005; 26: 1482	67	97%	94%	99%
	Meijboom *et al. JACC * 2006; 48: 1658	145	94%	98%	100%
	Ghostine *et al. * JACC 2006; 48: 1929	66	72%	99%	97%
	Ong *et al.* AHJ 2006; 151: 1323	134	80%	93%	94%
	Busch *et al. * Eur Radiol 2006	25	82%	95%	95%
	Schuijf *et al. * JACC 2006; 48: 2508	114	85%	97%	90%
	Miller *et al.* AHA Scientific Sessions 2007	291	85%	90%	83%

**Table 2.** Magnetic Resonance Angiography of the Coronary Arteries

Technique	Reference	# Patients	Sensitivity	Specificity
2D breathhold	Manning *et al.* NEJM 1993; 328: 828	39	90%	92%
	Pennell *et al.* Heart 1993; 70: 315	30	85%	NA
	Post *et al.* EHJ 1997; 18: 426	35	35%	63%
3D navigator, retrospective-gating	Woodard *et al.* AJR 1998; 170: 883	10	70%	NA
	Kessler *et al.* Radiology 1992; 210: 566	73	65%	88%
	Sandstede *et al.* AJR 1999; 172: 135	30	81%	89%
	Sardanelli *et al.* Radiology 2000; 214: 808	42	82%	89%
	Kim *et al.* NEJM 2001; 345: 1863	109	83%	42%
3D navigator, prospective-gating	Weber *et al.* Eur Radiol 2002; 12: 718	15	88%	94%
3D breathhold and/or contrast-enhanced	Regenfus *et al.* AJC 2002; 90: 725	50	94%	57%
	Van Guens *et al.* Radiology 2002; 217: 270	38	68%	97%
	Nikolaou *et al.* Eur Radiol 2002; 12: 1663	20	79%	70%
